# Cross-sectional Study of Factors Associated With Suicide Ideation in Ontario Adolescents

**DOI:** 10.1177/07067437221111364

**Published:** 2022-07-12

**Authors:** Jonah Rakoff, Jesus Chavarria, Hayley A. Hamilton, Tara Elton-Marshall

**Affiliations:** 1 Michael G. DeGroote School of Medicine, 3710 McMaster University, Hamilton, Ontario, Canada; 2 Department of Psychology, 7978 Western University, London, Ontario, Canada; 3 Institute for Mental Health Policy Research, Centre for Addiction and Mental Health, Toronto, Ontario, Canada; 4 Dalla Lana School of Public Health, University of Toronto, Toronto, Ontario, Canada; 5 School of Epidemiology and Public Health University of Ottawa, Ottawa, Ontario, Canada

**Keywords:** adolescence, epidemiology, Ontario, suicide

## Abstract

**Objective:**

Suicide is the second leading cause of death in Canadian adolescents. The Interpersonal Theory of Suicide attempts to explain suicide etiology and proposes that feelings of perceived burdensomeness or thwarted belongingness lead to suicide ideation, but this has not been extensively studied in adolescents. This study aimed to use the Interpersonal Theory of Suicide to examine factors that may be associated with suicide ideation in adolescents. The factors of interest were school connectedness, perceived availability of support, self-esteem, feelings of worthlessness, feelings of hopelessness, bullying and cyberbullying victimization, substance use, and social media use.

**Methods:**

Data were from the 2017 Ontario Student Drug Use and Health Survey, a survey of 7th to 12th graders enrolled in a publicly funded school in Ontario. Weighted multivariate logistic regression of suicide ideation on all exposure variables was conducted.

**Results:**

13.6% of students in the sample endorsed having suicidal ideation in the preceding 12 months. Not knowing where to turn to for support, feeling worthless, endorsing low self-esteem, being bullied, and using cannabis were each associated with greater odds of suicide ideation. Feeling hopeless, social media use, using alcohol and tobacco**,** and being cyberbullied were not associated with suicide ideation in the weighted multivariate logistic regression model.

**Conclusions:**

This study is consistent with the Interpersonal Theory of Suicide as low self-esteem and feelings of worthlessness, two indicators of perceived burdensomeness, and not knowing where to turn to for support, an indicator of thwarted belongingness, were associated with greater odds of suicide ideation. These findings can help guide interventions aimed at reducing the burden of suicidality during adolescence and demonstrate the need to provide accessible mental health support for youth.

## Abrégé

**Objectif:**

Le suicide est la deuxième cause de décès en importance des adolescents canadiens. La théorie interpersonnelle du suicide tente d’expliquer l’étiologie du suicide et propose que les sentiments de fardeau perçu ou d’appartenance frustrée mènent à l’idéation suicidaire, mais cela n’a pas été grandement étudié chez les adolescents. La présente étude visait à utiliser la théorie interpersonnelle du suicide pour examiner les facteurs qui peuvent être associés à l’idéation suicidaire chez les adolescents. Les facteurs d’intérêt étaient le sentiment d’appartenance à l’école, la disponibilité perçue du soutien, l’estime de soi, les sentiments d’inutilité, de désespoir, la victimisation d’intimidation et de cyberintimidation, l’utilisation de substances et l’utilisation des médias sociaux.

**Méthodes:**

Les données étaient tirées de l’Enquête de 2017 sur la consommation de drogues et la santé des élèves de l’Ontario, une enquête menée auprès d’élèves de la 7^e^ à la 12^e^ année inscrits dans une école publique de l’Ontario. La régression logistique multivariée pondérée de l’idéation suicidaire a été menée pour toutes les variables de l’exposition.

**Résultats:**

Chez les élèves de l’échantillon, 13,6% confirmaient avoir une idéation suicidaire, dans les 12 mois précédents. Ne pas savoir où demander du soutien, se sentir sans valeur, déplorer une faible estime de soi, être intimidé et utiliser du cannabis étaient tous associés à de plus grandes probabilités d’idéation suicidaire. Se sentir désespéré, utiliser les médias sociaux, consommer alcool et tabac, et être cyber intimidé n’étaient pas associés à l’idéation suicidaire dans le modèle de régression logistique multivariée pondérée.

**Conclusions:**

La présente étude correspond à la théorie interpersonnelle du suicide car la faible estime de soi et les sentiments d’être sans valeur, deux indicateurs du fardeau perçu, et ne pas savoir où demander de l’aide, un indicateur de l’appartenance frustrée, étaient associés à de plus fortes probabilités d’idéation suicidaire. Ces résultats peuvent aider à guider les interventions visant à réduire la charge de la suicidabilité durant l’adolescence et à démontrer le besoin d’offrir aux jeunes des soutiens accessibles de la santé mentale.

## Introduction

Suicide has been the second leading cause of death annually in Canada among males and females aged 15–24 since 2000, accounting for approximately one-quarter of all deaths in this age group.^1,2^ For every adolescent completed suicide, it is estimated that there are 50 to 100 attempted suicides.^3^ Given these concerning statistics, it is vital to understand the risk factors that lead to suicidal ideation among adolescents.

The Interpersonal Theory of Suicide^4^ attempts to explain suicide etiology and has garnered empirical support.^5^ The theory hypothesizes that perceived burdensomeness (i.e., the belief that one's existence is a burden to others and/or to society) and thwarted belongingness (i.e., when the psychological need to belong is unmet) are independent and sufficient causes of passive suicide ideation,^4^ with both consisting of two subordinate factors. Thwarted belongingness consists of loneliness, which is the cognitive belief that one has too few social connections, and the absence of reciprocally-caring relationships, which provide support and are characterized by positive feelings. Perceived burdensomeness consists of the belief that one is a liability to others, and cognitions of self-hatred. Suicidal ideation transforms into suicidal desire when an individual experiences a thwarted need to belong and perceived burdensomeness simultaneously, and is hopeless about these psychological states, perceiving them as stable and unchanging. For suicidal desire to result in suicidal behavior, an individual must acquire the capability for suicide through a reduced fear of death and increased tolerance to physical pain.^4^ The Interpersonal Needs Questionnaire was developed to empirically study the etiology of suicidal desire and behavior based on the theory and has been validated in adults and undergraduate students.^6^

While the Interpersonal Theory is hypothesized to apply across the lifespan,^7^ there are few studies that test its validity among adolescent samples. Numerous aspects of adolescence appear to align with the theory and may highlight potential risk factors for suicidal ideation. For instance, low school connectedness and a lack of social support are indicators of thwarted belongingness, whereas low self-esteem and feelings of worthlessness are indicators of perceived burdensomeness.^4,8^ Bullying or cyberbullying may also result in both thwarted belongingness and perceived burdensomeness^9^ as victims may feel socially isolated and unwanted, with the need for support being perceived as a burden on others.^9^ Related, social media use is paradoxically associated with mental distress and suicidality among youth,^10^ but has the potential to provide support and decrease perceived loneliness.^11^ Substance use heightens the risk for suicidal behavior^12^ and may also act as a coping mechanism for the negative psychological states of thwarted belongingness and perceived burdensomeness.^13^ Although involvement with peer groups can be protective against suicidal ideation,^14^ involvement with deviant peer groups has been associated with an increased risk of suicidal ideation.^15^ Given the complicated relationships between these factors and suicidality, it is worthwhile to study how these factors contribute to suicide ideation in adolescents to better understand adolescent suicide etiology and ultimately prevent suicides.

Because of the relative paucity of research examining the Interpersonal Theory in adolescents compared with adults, the objective of the current study was to use the Interpersonal Theory to examine factors that may be associated with suicide ideation in a sample of adolescents. This study aimed to assess whether the hypotheses of the theory may apply to adolescents, as well as identify risk factors for suicide ideation. Specifically, we tested whether factors related to perceived burdensomeness and thwarted belongingness were associated with suicide ideation in a sample of adolescents enrolled in a publicly funded school in Ontario. We hypothesized each of the following eleven factors would be associated with increased odds of suicide ideation: lower school connectedness, lack of perceived availability of support, low self-esteem, feelings of worthlessness, feelings of hopelessness, bullying victimization, cyberbullying victimization, frequent social media use, tobacco use, alcohol use, and cannabis use.

## Methods

### Survey and Participants

This was a cross-sectional study and data were from the 2017 Ontario Student Drug Use and Health Survey (OSDUHS), a biennial survey with a target population of 7th to 12th graders enrolled in a publicly funded school in Ontario.^16^ Students who were homeschooled, institutionalized, and enrolled in private schools, schools in First Nations communities, or schools in the remote northern region of Ontario were not sampled. The target population represents an estimated 91% of all Ontario adolescents aged 12–18 years.^16^ The survey was administered between November 2016 and June 2017.

The 2017 OSDUHS used a stratified, two-stage cluster sampling design. Within each of 18 regional strata, eligible schools were selected with a probability proportionate-to-size selection so that larger schools had a greater probability of being selected. Within each selected school, one or two classes per grade were randomly selected with equal probability. All students in the selected class with a signed consent form were eligible to participate.^16^

In 2017, 214 schools and 764 classes participated in the survey. A total of 18,773 students were enrolled in the participating classes. Of these students, 11,596 participated in the survey and 11,435 cases (61%) were considered completions after data quality criteria were applied.^16^ Reasons for non-participation included absenteeism, unreturned consent forms, or parental refusal.^16^

In each participating classroom, two different forms of the OSDUHS questionnaire were equally distributed. The forms were distributed alternately to achieve near-equal random samples completing each form. One of these forms included the question of suicide ideation. A total of 6364 students completed the form that included this question.^16^ Of these students, 5912 responded to the question on suicide ideation and the total sample size for the current study was therefore 5912 subjects.

### Variables

#### Demographic Variables

Participants reported sex at birth, grade, race/ethnicity, and socioeconomic status. Eight categories of race/ethnicity were created based on participants’ self-reported background: White, Black, East/Southeast Asian, South Asian, West Asian/Arab, Latino, Indigenous, and Multiracial. Socioeconomic status was reported by selecting a ring on a ladder based on where they thought their family would be out of 10 levels, with the top of the ladder described as people who are the “best off” and the bottom described as people who are the “worst off.”^17^ Region of Ontario was included as a demographic variable but was not self-reported by participants. Regions were Greater Toronto Area (GTA), North, West, and East.

#### Suicide Ideation

Suicide ideation was measured by the response to the question “in the last 12 months, did you ever seriously consider attempting suicide?” Response options were “Yes” and “No.”

#### School Connectedness

The degree of school connectedness was measured by two questions: “I feel close to people at school” and “I feel like I am part of this school.” Response options for each question were “Strongly agree,” “Somewhat agree,” “Somewhat disagree,” and “Strongly disagree.” Responses to each question were separately scored ranging from 0 for “Strongly agree” to 3 for “Strongly disagree.” Scores from the two questions were then added together to give an overall score ranging from 0 to 6, with a higher score indicating lower school connectedness.

#### Perceived Availability of Support

Perceived availability of support was measured by the question “In the last 12 months, was there a time when you wanted to talk to someone about a mental health or emotional problem you had, but did not know where to turn?” Response options were “Yes” and “No.”

#### Self-Esteem

The question related to self-esteem in the OSDUHS was a global measure of self-esteem from the Rosenberg Self-Esteem Scale.^18^ Participants were asked “How much do you agree or disagree with the following statement: on the whole, I am satisfied with myself.” Response options were “Strongly agree,” “Somewhat agree,” “Somewhat disagree,” and “Strongly disagree.”

#### Feelings of Worthlessness and Hopelessness

The questions related to feelings of worthlessness and hopelessness were included in the OSDUHS as part of the Kessler 6-item Psychological Distress Scale.^19^ Feelings of worthlessness and hopelessness were respectively measured by the questions “In the last 4 weeks, about how often did you feel worthless?” and “In the last 4 weeks, about how often did you feel hopeless?” Response options were “None of the time,” “A little of the time,” “Some of the time,” “Most of the time,” and “All of the time.”

#### Bullying and Cyberbullying

Bullying victimization was measured by the question “Since September, how often have you been bullied at school?” Response options were “Was not bullied,” “Daily or almost daily,” “About once a week,” “About once a month,” and “Less than once a month.” Cyberbullying victimization was measured by the question “in the last 12 months, how often did other people bully or pick on you electronically or through the Internet?” Response options were “Don't use the Internet or a cellphone,” “Never,” “Once,” “2 or 3 times,” and “4 or more times.”

#### Substance use

Tobacco use, alcohol use and cannabis use were respectively assessed by the following questions: “in the last 12 months, how often did you smoke tobacco cigarettes?” “in the last 12 months, how often did you drink alcohol?” and “in the last 12 months, how often did you use cannabis?” Tobacco use was grouped as daily use and non-daily use/non-use. Alcohol use was grouped as non-use, occasional use (monthly or less), and regular use (at least twice a month). Cannabis use was grouped as no use, 1 or 2 times, 3 to 5 times, 6 to 19 times, and 20 or more times.

#### Social Media Use

Social media use was measured by the question “About how many hours a day do you usually spend on social media sites or apps, such as Instagram, Snapchat, Twitter, Facebook, Ask.fm, either posting or browsing?” Response options were “Less than 1 h a day,” “About 1 h a day,” “2 h a day,” “3 to 4 h a day,” “5 to 6 h a day,” “7 or more hours a day,” “Use social media, but not every day,” “Use the Internet, but don't use social media,” and “Don't use the Internet.” Responses were grouped as social media and internet non-users/non-daily users, 1 h or less a day, 2 h a day, 3 or 4 h a day, and 5 or more hours a day.

### Data Analysis

The total number of responses to each question was calculated for reference to the total sample size. Analyses were weighted to account for the sampling design. The weighting accounted for the probability of selection, stratification, and clustering, and a poststratification weight was applied to restore the population sex distribution according to grade. Weighted bivariate analyses were conducted by applying a corrected Pearson chi-square test to two-way tables.^20^ Weighted multivariate logistic regression of suicide ideation on all exposure variables was conducted and outcomes were reported as odds ratios. Five demographic variables were included as potential covariates: sex, grade, region, ethnicity, and socioeconomic status.^16^ Exposure variables were selected prior to any analyses examining potential associations and there was no addition or removal of variables to or from the model following the analysis. Joint-hypothesis tests were performed for each categorical variable using an adjusted Wald test to determine overall significance and are reported along with the individual significance of each category. To account for multiple hypothesis testing of the Wald test for the eleven variables of interest, a Bonferroni correction was used and a *p*-value less than 0.0045 was considered statistically significant. Potential multicollinearity was assessed using the variance inflation factor for each exposure variable, which measures how much the variance of a regression coefficient depends on its correlation with other independent variables,^21^ and greater than 5 was considered indicative of collinearity.^21^ Stata 16.0 was used for all analyses using the SVY command for complex survey data.^22^

## Results

### Prevalence of Responses

Sample characteristics are presented in Table 1. Overall, 794 students (13.6%) responded that they had seriously contemplated suicide in the last 12 months.

**Table 1.** Sample Characteristics and Bivariate Analysis Examining Factors Associated with Suicide Ideation among Adolescents in Ontario (OSDUHS, 2017).

	Suicide ideation *n* = 794	No suicide ideation *n* = 5118	Corrected Pearson Chi-Square Test statistic
	*n* (column weighted %)	*n* (column weighted %)	
Sex			17.9 (*p *< 0.001)
Female	583 (68.1)	2802 (45.7)	
Male	211 (31.9)	2316 (54.3)	
Total	794	5118	(Missing = 0)
Grade			2.4 (*p* = 0.081)
7	67 (7.6)	797 (12.1)	
8	131 (9.7)	840 (11.5)	
9	141 (17.5)	1014 (17.1)	
10	152 (19.7)	913 (17.5)	
11	134 (15.3)	784 (19.2)	
12	168 (30.3)	761 (22.6)	
Total	793	5109	(Missing = 10)
Ethnicity			2.4 (*p* = 0.073)
White	419 (46.7)	2940 (54.4)	
Black	70 (13.7)	370 (12.2)	
East Asian/Southeast Asian	55 (5.3)	339 (6.5)	
South Asian	44 (4)	346 (6.1)	
West Asian/Arab	27 (7.4)	224 (7)	
Latino	13 (5.5)	86 (2.1)	
Indigenous	13 (0.7)	41 (0.5)	
Multiracial	121 (16.8)	543 (11.2)	
Total	762	4889	(Missing = 261)
Region			1.3 (*p* = 0.28)
GTA	359 (58.1)	2163 (55.2)	
North	111 (5.7)	779 (6.3)	
West	114 (20.6)	647 (18.7)	
East	210 (15.7)	1529 (19.8)	
Total	794	5118	(Missing = 0)
Mean family socioeconomic status (SD)	6.2 (0.1)	7 (0.05)	12.5 (*p *< 0.001) (Missing = 142)
Mean school connectedness (SD)	2.5 (0.12)	1.4 (0.05)	107.1 (*p *< 0.001) (Missing = 76)
Wanted to talk about a mental health problem			228.1 (*p *< 0.001)
Did not know where to turn	551 (72.5)	1253 (25)	
Knew where to turn	241 (27.5)	3839 (75)	
Total	792	5092	(Missing = 28)
Satisfied with self			125.8 (*p *< 0.001)
Strongly disagree	230 (29.2)	183 (3.6)	
Somewhat disagree	306 (38.8)	647 (12.8)	
Somewhat agree	210 (26.6)	2404 (47.4)	
Strongly agree	43 (5.4)	1835 (36.2)	
Total	789	5069	(Missing = 54)
Feel worthless			62.9 (*p *< 0.001)
All of the time	175 (22.1)	90 (1.8)	
Most of the time	203 (25.6)	196 (3.8)	
Some of the time	199 (25.1)	474 (9.3)	
A little of the time	134 (16.9)	869 (17)	
None of the time	82 (10.3)	3477 (68.1)	
Total	793	5106	(Missing = 13)
Feel hopeless			65.2 (*p *< 0.001)
All of the time	127 (16.1)	74 (1.5)	
Most of the time	205 (25.9)	237 (4.7)	
Some of the time	229 (29)	703 (13.8)	
A little of the time	158 (20)	1349 (26.5)	
None of the time	72 (9)	2721 (53.5)	
Total	791	5084	(Missing = 37)
Bullying victimization			28.7 (*p *< 0.001)
Daily	50 (6.5)	96 (1.1)	
Once a week	91 (14.0)	189 (3.1)	
Once a month	76 (7.8)	190 (2.8)	
Less than once a month	109 (15.2)	397 (8.7)	
Not bullied	461 (56.5)	4157 (84.3)	
Total	787	5029	(Missing = 96)
Cyberbullying Victimization			18.3 (*p *< 0.001)
4 or more times	112 (12.5)	154 (2.7)	
2 or 3 times	122 (14.2)	251 (5.5)	
Once	114 (13.3)	471 (8.8)	
Never	433 (60)	4158 (83)	
Total	781	5034	(Missing = 97)
Smoke cigarettes daily			11.4 (*p *< 0.001)
Yes	45 (7.1)	80 (1.8)	
No	747 (92.9)	5029 (93.3)	
Total	792	5109	(Missing = 11)
Drink alcohol			9.8 (*p *< 0.001)
Regular use	154 (21.4)	542 (12.1)	
Occasional use	281 (34.9)	1372 (28.3)	
No use	357 (43.7)	3185 (59.6)	
Total	792	5099	(Missing = 21)
Smoke cannabis			11.1 (*p *< 0.001).
20 or more times	68 (10.8)	189 (4.3)	
6 to 19 times	68 (7.6)	179 (4)	
3 to 5 times	48 (7.1)	131 (3.1)	
1 or 2 times	64 (9.1)	265 (5.4)	
None	539 (65.3)	4300 (83.2)	
Total	787	5064	(Missing = 61)
Hours of social media per day			8.7 (*p *< 0.001)
5 or more	271 (34.1)	847 (17.9)	
3 or 4	208 (24.3)	1249 (25.7)	
2	125 (16.2)	1054 (19.6)	
1 or less	111 (13.7)	1176 (23.0)	
Do not use or do not use daily	77 (11.7)	774 (13.8)	
Total	792	5100	(Missing = 20)

*Note.* Cell totals do not add up to the same value due to missingness across variables.

### Associations

The results of the bivariate analyses are presented in Table 1. The Pearson chi-square test statistic for each exposure variable was significant (*p *< 0.001).

The results of the weighted multivariate logistic regression are presented in Table 2. The thwarted belongingness indicator of school connectedness was not associated with suicide ideation whereas not knowing where to turn to talk about a mental health problem was associated with an increased likelihood of suicide ideation. The perceived burdensomeness indicators of endorsing low self-esteem and feelings of worthlessness were both associated with suicide ideation. Feelings of hopelessness and social media use were not associated with suicide ideation. Bullying victimization was associated with suicide ideation while cyberbullying was not associated with suicide ideation. For the substance use variables, cannabis use was associated with suicide ideation while alcohol use and daily cigarette smoking were not associated with suicide ideation.

**Table 2.** Weighted Logistic Regression Analysis Examining Factors Associated with Suicide Ideation among Adolescents in Ontario (OSDUHS, 2017).

	Weighted multivariate OR (95% CI)	*p-*Value	Global test of significance F statistic
School connectedness	1.01 (0.85, 1.20)	0.93	0.01 (*p* = 0.93)
Wanted to talk about a mental health problem			17.3 (*p *< 0.001)
Did not know where to turn	1.99 (1.43, 2.75)	< 0.001	
Knew where to turn	(Ref)		
Self-esteem (satisfied with self)			22.2 (*p *< 0.001)
Strongly disagree	5.63 (2.19, 14.48)	< 0.001	
Somewhat disagree	3.33 (1.38, 8.03)	0.008	
Somewhat agree	1.29 (0.64, 2.62)	0.475	
Strongly agree	(Ref)		
Feel worthless			12.5 (*p *< 0.001)
All of the time	7.89 (3.26, 19.11)	< 0.001	
Most of the time	11.93 (5.52, 25.8)	< 0.001	
Some of the time	5.49 (3.04, 9.94)	< 0.001	
A little of the time	3.73 (2.29, 6.07)	< 0.001	
None of the time	(Ref)		
Bullying victimization			4.8 (*p* = 0.0011)
Daily	2.34 (1.04, 5.24)	0.04	
Once a week	2.97 (1.51, 5.86)	0.002	
Once a month	1.58 (0.92, 2.72)	0.097	
Less than once a month	1.54 (0.88, 2.67)	0.13	
Not bullied	(Ref)		
Cyberbullying victimization			1.42 (*p* = 0.24)
4 or more times	1.53 (0.78, 3.0)	0.22	
2 or 3 times	1.26 (0.73, 2.19)	0.40	
Once	1.45 (1.0, 2.11)	0.051	
Never	(Ref)		
Feel hopeless			2.2 (*p* = 0.074)
All of the time	1.83 (0.37, 9.02)	0.78	
Most of the time	2.47 (0.96, 6.36)	0.06	
Some of the time	1.06 (0.37, 3.02)	0.91	
A little of the time	1.11 (0.55, 2.22)	0.78	
None of the time	(Ref)		
Smoke cigarettes daily			1.29 (*p* = 0.26)
Yes	1.68 (0.68, 4.12)	0.26	
No	(Ref)		
Drink alcohol			4.1 (*p* = 0.018)
Regular use	1.67 (0.97, 2.87)	0.064	
Occasional use	2.04 (1.26, 3.30)	0.004	
No use	(Ref)		
Smoke cannabis			5.2 (*p *< 0.001)
20 or more times	1.61 (0.88, 2.97)	0.12	
6 to 19 times	2.80 (1.65, 4.76)	< 0.001	
3 to 5 times	1.88 (0.97, 3.65)	0.062	
1 or 2 times	1.07 (0.64, 1.77)	0.80	
None	(Ref)		
Hours of social media per day			1.9 (*p* = 0.11)
5 or more	0.66 (0.33, 1.32)	0.24	
3 or 4	0.51 (0.29, 0.89)	0.019	
2	0.59 (0.31, 1.14)	0.12	
1 or less	0.83 (0.46, 1.47)	0.51	
Do not use or do not use daily	(Ref)		
Sex			1.39 (*p* = 0.24)
Female	1.32 (0.83, 2.10)	0.24	
Male	(Ref)		
Grade			2.3 (*p* = 0.05)
7	(Ref)		
8	1.29 (0.75, 2.21)	0.36	
9	1.29 (0.67, 2.48)	0.44	
10	0.96 (0.51, 1.80)	0.89	
11	0.54 (0.25, 1.14)	0.11	
12	0.78 (0.40, 1.54)	0.48	
Ethnicity			0.7 (*p* = 0.64)
White	(Ref)		
Black	1.88 (1.02, 3.46)	0.042	
East Asian/Southeast Asian	1.19 (0.67, 2.12)	0.55	
South Asian	1.04 (0.52, 2.07)	0.91	
West Asian/Arab	1.12 (0.42, 2.98)	0.82	
Latino	1.31 (0.66, 2.60)	0.44	
Indigenous	0.86 (0.26, 2.78)	0.80	
Multiracial	1.18 (0.78, 1.78)	0.43	
Region			1.7 (*p* = 0.17)
GTA	(Ref)		
North	0.98 (0.62, 1.55)	0.92	
West	0.81 (0.54, 1.22)	0.30	
East	0.71 (0.51, 0.99)	0.044	
Family socioeconomic status	0.94 (0.86, 1.02)	0.15	2.13 (*p* = 0.15)

### Variance Inflation Factors

The variance inflation factors are presented in Table 3 and was less than 5 for each variable.

**Table 3.** Variance Inflation Factors for Exposure Variables.

Variable	Variance inflation factor
School connectedness	1.29
Perceived availability of support	1.38
Self-esteem	1.88
Feelings of worthlessness	2.55
Bullying victimization	1.32
Cyberbullying victimization	1.31
Feelings of hopelessness	2.46
Tobacco use	1.13
Alcohol use	1.66
Cannabis use	1.55
Social media use	1.20
Mean variance inflation factor	1.47

## Discussion

With rates of suicide ideation on the rise^23^ it is critical to identify factors associated with a greater risk of suicide ideation. Using the Interpersonal Theory as a framework, this study investigated whether factors related to perceived burdensomeness and thwarted belongingness were associated with suicidal ideation among a sample of Ontario adolescents enrolled in publicly funded schools. Consistent with our hypotheses, we determined that not knowing where to turn for support, feeling worthless, having low self-esteem, being bullied, and using cannabis were associated with greater odds of suicide ideation. While feeling hopeless, being cyberbullied, using alcohol and tobacco, and social media use were associated with greater odds of suicide ideation in the bivariate analyses, these variables were unexpectedly not associated with suicide ideation in the multivariate model.

The perceived burdensomeness indicators of low self-esteem and feelings of worthlessness had the strongest association with suicide ideation. This is consistent with research highlighting the association between low self-esteem and suicide attempts and ideation among adolescents,^24^ and supports the Interpersonal Theory in adolescents. One explanation is that self-esteem becomes more important as an individual enters adolescence because the self is perceived as more independent.^24^ Self-esteem is thought to remain stable throughout adolescence, so low self-esteem may be a stable problem that leads to emotional distress.^24^ Assessing self-esteem as part of suicide screening, and interventions aimed at improving self-esteem in adolescents may therefore be important parts of suicide prevention programs.

Consistent with previous research that found social support to prospectively protect against suicide attempts,^7^ the thwarted belongingness indicator of perceived lack of availability of support, which represents the absence of reciprocally-caring relationships, was associated with increased odds of suicide ideation. This finding supports the Interpersonal Theory and suggests that mental health support for adolescents and awareness of how to access support are urgently needed. While persistent feelings of hopelessness were not associated with suicide ideation in the multivariate analysis, the Interpersonal Theory hypothesizes that hopelessness regarding the feelings of thwarted belongingness and perceived burdensomeness causes active suicidal desire, rather than hopelessness being an independent cause of suicidal ideation.^4^ However, this finding is not in accordance with the literature on the association between hopelessness and suicide, possibly because of the additional variables included in our model. Beck et al. developed the Beck Hopelessness Scale and found hopelessness to be significantly related to suicidal ideation,^25^ and the Beck Hopelessness Scale has also shown predictive validity for suicide ideation in adolescents.^26^

While social media use was associated with suicide ideation at the bivariate level, after controlling for other factors in the multivariate model, social media use was not significantly associated with suicide ideation. The literature on the association between social media use and depression and suicide is conflicting. Some evidence suggests that social media may provide a sense of connectedness for adolescents and decrease feelings of loneliness, as higher levels of Facebook social connectedness were found to be related to lower levels of depression and anxiety^27^ and posting status updates on Facebook was found to decrease loneliness by feeling more connected with friends.^28^ Furthermore, social support and feelings of connectedness through social networks may be especially beneficial for those who have a harder time accessing support in person.^27^ However, other cross-sectional studies have found social media use to be associated with suicide attempts,^29^ and longitudinal studies are needed to better understand how social media use impacts suicidality in adolescents.

The finding that bullying is associated with suicide ideation is consistent with the literature on this topic^30^ and one study found that the association between bullying and suicide ideation in an adolescent sample was fully mediated by thwarted belongingness and perceived burdensomeness.^31^ Shneidman's psychache theory of suicide proposes that suicide results when psychological pain, characterized by guilt, loneliness, and other negative feelings, becomes unbearable^4^ and Bao, Li, Song, and Jiang similarly found that the association between bullying victimization and suicide ideation was fully mediated by psychological pain.^32^ In the same study, family togetherness and peer support decreased the effect of psychological pain on suicide ideation.^32^ Cyberbullying victimization is a known risk factor for suicide ideation^33^ but was not significantly associated with increased odds of suicide ideation in the multivariate model, possibly because the association is also mediated by the variables related to thwarted belongingness and perceived burdensomeness. Ameliorating feelings of psychological pain, thwarted belongingness, and perceived burdensomeness in bully victims may therefore offer a potential target for interventions, and increasing social support may be one method of reducing the effect of these feelings on the development of suicide ideation.

Substance use is another known risk factor that is associated with adolescent suicidal ideation.^34,35^ The increased risk of suicide ideation in daily cannabis users has been shown to be partly due to higher levels of perceived burdensomeness and thwarted belongingness.^36^ This supports the idea that those who use substances may perceive themselves to be a burden on others and have a reduced sense of belonging which contributes to their suicidality. Biological theories of suicide propose that dysregulation of the serotonergic, noradrenergic, and dopaminergic neurotransmitter systems contribute to suicidal thoughts and behavior,^37^ and animal studies have found cannabis use in adolescence to produce dysfunction in each of these neurotransmitter systems.^38^ While alcohol use was not significantly associated with suicide ideation in the multivariate model, Lamis and Malone found that perceived burdensomeness and thwarted belongingness were mediators between alcohol-related problems and suicidal proneness in college students,^39^ which may account for the lack of a direct association between alcohol use and suicide ideation. Assessing substance use may therefore be another important aspect of suicide screening in adolescents, and adolescent cannabis users may be a specific group to target for suicide prevention programs.

There are several limitations of the study. First, the study is cross-sectional, so causality, temporality and directionality of associations cannot be inferred. The study was also limited to using a single item asking about suicide ideation and could not differentiate the severity of the ideation. Additionally, there were different time intervals for some of the variables, further limiting any inference of temporality or directionality. While the factors that were associated with suicide ideation were consistent with the Interpersonal Theory, it is not possible to determine whether these factors contributed to suicide ideation or occurred before an individual experienced suicide ideation. The study design and use of OSDUHS did, however, allow for a large sample size with relatively reliable data collection. Second, OSDUHS did not use the Interpersonal Needs Questionnaire to guide the selection of items included in the survey and the variables selected in this study are proxy measures for thwarted belongingness and perceived burdensomeness. However, the constructs of thwarted belongingness and perceived burdensomeness are general and the items in OSDUHS used in this study align with the items in the Interpersonal Needs Questionnaire. Third, the OSDUHS target population is adolescents enrolled in public schools and excluded groups include students who were homeschooled, institutionalized, and enrolled in private schools, schools in First Nations communities, or schools in the remote northern region of Ontario. Some of the excluded groups, such as those who are marginally housed, incarcerated, or in group homes, experience a greater burden of disease related to suicide and suicidality,^40,41^ and the findings from the current study may not be generalizable to these groups. The target population is, however, representative of the vast majority of Ontario children and adolescents.^16^ Additionally, the results are not generalizable to suicide attempts in adolescents as this study did not assess whether the factors associated with increased odds of suicide ideation were associated with suicidal attempts.

## Conclusion

Not knowing where to turn for support, feeling worthless, having low self-esteem, being bullied, and using cannabis were associated with greater suicide ideation in this sample of 7th to 12th graders enrolled in a publicly funded school in Ontario. The associations of not knowing where to turn to for support, feeling worthless, and having low self-esteem with suicide ideation may lend support to the validity of the Interpersonal Theory of Suicide in adolescents as thwarted belongingness and perceived burdensomeness may result in suicide ideation. The findings also demonstrate the urgent need to provide accessible mental health support for youth in Ontario, particularly for the high-risk groups identified.

## Supplemental Material

sj-docx-1-cpa-10.1177_07067437221111364 - Supplemental material for Cross-sectional Study of Factors Associated With Suicide Ideation in Ontario Adolescents Click here for additional data file. Supplemental material, sj-docx-1-cpa-10.1177_07067437221111364 for Cross-sectional Study of Factors Associated With Suicide Ideation in Ontario Adolescents by Jonah Rakoff, Jesus Chavarria and Hayley A. Hamilton, Tara Elton-Marshall in The Canadian Journal of Psychiatry
