# Supplementary material for: Cross-sectional Study of Factors Associated With Suicide Ideation in Ontario Adolescents
Source: Can J Psychiatry. 2022 Jul 12;68(5):327–37. doi: 10.1177/07067437221111364 (PMC10192826; doi:10.1177/07067437221111364)
Supplement: sj-docx-1-cpa-10.1177_07067437221111364 - Supplemental material for Cross-sectional Study of Factors Associated With Suicide Ideation in Ontario Adolescents [file sj-docx-1-cpa-10.1177_07067437221111364.docx]

STROBE Statement—checklist of items that should be included in reports of observational studies

|  | Item No. | Recommendation | Page  No. | Relevant text from manuscript |
| --- | --- | --- | --- | --- |
| **Title and abstract** | 1 | (*a*) Indicate the study’s design with a commonly used term in the title or the abstract | 1 |  |
|  |  | (*b*) Provide in the abstract an informative and balanced summary of what was done and what was found | 2 |  |
| Introduction | | | |  |
| Background/rationale | 2 | Explain the scientific background and rationale for the investigation being reported | 3–4 | - The Interpersonal Theory of Suicide attempts to explain suicide etiology and has garnered empirical support… - While the theory is hypothesized to apply across the lifespan, there are few studies that test its validity among adolescent samples… it is worthwhile to study how these factors contribute to suicide ideation in adolescents to better understand adolescent suicide etiology and ultimately prevent suicides. |
| Objectives | 3 | State specific objectives, including any prespecified hypotheses | 4–5 | - Because of the relative paucity of research examining the Interpersonal Theory of Suicide in adolescents compared with adults, the objective of the current study was to use the Interpersonal Theory to examine factors that may be associated with suicide ideation in a sample of adolescents. - We hypothesized each of the following eleven factors would be associated with increased odds of suicide ideation: lower school connectedness, lack of perceived availability of support, low self-esteem, feelings of worthlessness, feelings of hopelessness, bullying victimization, cyberbullying victimization, frequent social media use, tobacco use, alcohol use, and cannabis use. |
| Methods | | | |  |
| Study design | 4 | Present key elements of study design early in the paper | 5 | - This was a cross-sectional study and data were from the 2017 Ontario Student Drug Use and Health Survey |
| Setting | 5 | Describe the setting, locations, and relevant dates, including periods of recruitment, exposure, follow-up, and data collection | 5 | - target population of 7th to 12th graders enrolled in a publicly funded school in Ontario - The survey was administered in the sampled classrooms during class periods between November 2016 and June 2017. |
| Participants | 6 | *Cross-sectional study*—Give the eligibility criteria, and the sources and methods of selection of participants | 5 | - Within each of 18 regional strata, eligible schools were selected with a probability proportionate-to-size selection so that larger schools had a greater probability of being selected. Within each selected school, one or two classes per grade was randomly selected with equal probability. All students in the selected class with a signed consent form were eligible to participate |
| Variables | 7 | Clearly define all outcomes, exposures, predictors, potential confounders, and effect modifiers. Give diagnostic criteria, if applicable | 6–8 |  |
| Data sources/ measurement | 8* | For each variable of interest, give sources of data and details of methods of assessment (measurement). Describe comparability of assessment methods if there is more than one group | 6–8 |  |
| Bias | 9 | Describe any efforts to address potential sources of bias | 8–9 | - Analyses were weighted to account for the sampling design used in OSDUHS. - Five demographic variables were included as potential covariates: sex, grade, region, ethnicity, and socioeconomic status - Independent variables were selected prior to any analyses examining potential associations and there was no addition or removal of variables to or from the model following the analysis - To account for multiple hypothesis testing of the Wald test for the eleven variables of interest, a Bonferroni correction was used and a *p* value less than 0.0045 was considered statistically significant - Risk of collinearity was assessed using the variance inflation factor for each exposure variable |
| Study size | 10 | Explain how the study size was arrived at | 6 | - In each participating classroom, two different forms of the OSDUHS questionnaire were equally distributed. The forms were distributed alternately to achieve near-equal random samples completing each form. One of these forms included the question on suicide ideation. A total of 6364 students completed the form that included this question^16^. Of these students, 5912 responded to the question on suicide ideation and the total sample size for the current study was therefore 5912 subjects. |

| Quantitative variables | 11 | Explain how quantitative variables were handled in the analyses. If applicable, describe which groupings were chosen and why | 6–8 |  |
| --- | --- | --- | --- | --- |
| Statistical methods | 12 | (*a*) Describe all statistical methods, including those used to control for confounding | 8–9 |  |
|  |  | (*b*) Describe any methods used to examine subgroups and interactions | N/A |  |
|  |  | (*c*) Explain how missing data were addressed | 8 | - The total number of responses to each question were calculated for reference to the total sample size |
|  |  | (*d*) *Cross-sectional study*—If applicable, describe analytical methods taking account of sampling strategy | 9 | - Stata 16.0 was used for all analyses using the SVY command for complex survey data |
|  |  | (*e*) Describe any sensitivity analyses | N/A |  |
| Results | | | | |
| Participants | 13* | (a) Report numbers of individuals at each stage of study—eg numbers potentially eligible, examined for eligibility, confirmed eligible, included in the study, completing follow-up, and analysed | 6 | - A total of 18,773 students were enrolled in the participating classes. Of these students, 11,596 participated in the survey and 11,435 cases (61%) were considered completions after data quality criteria were applied - In each participating classroom, two different forms of the OSDUHS questionnaire were equally distributed. The forms were distributed alternately to achieve near-equal random samples completing each form. One of these forms included the question on suicide ideation. A total of 6364 students completed the form that included this question. Of these students, 5912 responded to the question on suicide ideation and the total sample size for the current study was therefore 5912 subjects. |
|  |  | (b) Give reasons for non-participation at each stage | 6 | - Reasons for non-participation included absenteeism, unreturned consent forms, or parental refusal |
|  |  | (c) Consider use of a flow diagram |  |  |
| Descriptive data | 14* | (a) Give characteristics of study participants (eg demographic, clinical, social) and information on exposures and potential confounders | 22–26 | Table 1 |
|  |  | (b) Indicate number of participants with missing data for each variable of interest | 22–26 | Table 1 |
| Outcome data | 15* | *Cross-sectional study—*Report numbers of outcome events or summary measures | 9 | - 794 students (13.6%) responded that they had seriously contemplated suicide in the last 12 months. |
| Main results | 16 | (*a*) Give unadjusted estimates and, if applicable, confounder-adjusted estimates and their precision (eg, 95% confidence interval). Make clear which confounders were adjusted for and why they were included | 22–30 | Table 2 |
|  |  | (*b*) Report category boundaries when continuous variables were categorized | N/A |  |
|  |  | (*c*) If relevant, consider translating estimates of relative risk into absolute risk for a meaningful time period | N/A |  |

| Other analyses | 17 | Report other analyses done—eg analyses of subgroups and interactions, and sensitivity analyses | N/A |  |
| --- | --- | --- | --- | --- |
| Discussion | | | | |
| Key results | 18 | Summarise key results with reference to study objectives | 10–11 | - we determined that not knowing where to turn to for support, feeling worthless, having low self-esteem, being bullied, and using cannabis was associated with greater odds of suicide ideation. While feeling hopeless, being cyberbullied, using alcohol and tobacco, and social media use were associated with greater odds suicide ideation in the bivariate analyses, these variables were unexpectedly not associated with suicide ideation in the multivariate model. |
| Limitations | 19 | Discuss limitations of the study, taking into account sources of potential bias or imprecision. Discuss both direction and magnitude of any potential bias | 14–15 |  |
| Interpretation | 20 | Give a cautious overall interpretation of results considering objectives, limitations, multiplicity of analyses, results from similar studies, and other relevant evidence | 11–15 |  |
| Generalisability | 21 | Discuss the generalisability (external validity) of the study results | 14–15 | - The OSDUHS target population is adolescents enrolled in public school and excluded groups include students who were homeschooled, institutionalized, and enrolled in private schools, schools in First Nations communities, or schools in the remote northern region of Ontario. Some of the excluded groups, such as those who are marginally housed, incarcerated, or in group homes, experience a greater burden of disease related to suicide and suicidality, and the findings from the current study may not be generalizable to these groups - Additionally, the results from this study are not generalizable to suicide attempts and behaviour in adolescents as this study did not assess whether the factors associated with increased odds of suicide ideation were associated with a suicidal attempt in the sample. |
| Other information | |  | | |
| Funding | 22 | Give the source of funding and the role of the funders for the present study and, if applicable, for the original study on which the present article is based | 16 | - This research received no specific grant from any funding agency in the public, commercial, or not-for-profit sectors. |

*Give information separately for cases and controls in case-control studies and, if applicable, for exposed and unexposed groups in cohort and cross-sectional studies.
